# A novel approach to improve poly-γ-glutamic acid production by NADPH Regeneration in *Bacillus licheniformis* WX-02

**DOI:** 10.1038/srep43404

**Published:** 2017-02-23

**Authors:** Dongbo Cai, Penghui He, Xingcheng Lu, Chengjun Zhu, Jiang Zhu, Yangyang Zhan, Qin Wang, Zhiyou Wen, Shouwen Chen

**Affiliations:** 1 Hubei Collaborative Innovation Center for Green Transformation of Bio-Resources, College of Life Sciences, Hubei University, Wuhan 430062, China; 2 College of Food Science and Technology, Huazhong Agricultural University, Wuhan 430070, China; 3 Department of Food Science and Human Nutrition, Iowa State University, Ames, Iowa 50011, United States

## Abstract

Poly-γ-glutamic acid (γ-PGA) is an important biochemical product with a variety of applications. This work reports a novel approach to improve γ-PGA through over expression of key enzymes in cofactor NADPH generating process for NADPH pool. Six genes encoding the key enzymes in NADPH generation were over-expressed in the γ-PGA producing strain *B. licheniformis* WX-02. Among various recombinants, the strain over-expressing *zwf* gene (coding for glucose-6-phosphate dehydrogenase), WX-zwf, produced the highest γ-PGA concentration (9.13 g/L), 35% improvement compared to the control strain WX-pHY300. However, the growth rates and glucose uptake rates of the mutant WX-zwf were decreased. The transcriptional levels of the genes *pgsB* and *pgsC* responsible for γ-PGA biosynthesis were increased by 8.21- and 5.26-fold, respectively. The Zwf activity of the *zwf* over expression strain increased by 9.28-fold, which led to the improvement of the NADPH generation, and decrease of accumulation of by-products acetoin and 2,3-butanediol. Collectively, these results demonstrated that NADPH generation via over-expression of Zwf is as an effective strategy to improve the γ-PGA production in *B. licheniformis*.

Poly-γ-glutamic acid (γ-PGA) is a multifunctional biopolymer consisting of D- and L-glutamate as monomers. Due to its features of cation chelating, hygroscopicity, water-solubility, non-toxicity and biodegradability, γ-PGA has been applied in cosmetics, medicine, food, water treatment, and agricultural industries, such as material and drug carrier, food preservative, metal ion chelating agents and highly water absorbable hydrogels^1^,^2^,^3^.

γ-PGA is commonly produced through bacterial transformation of glutamate. In general, *Bacillus* strains were the major γ-PGA producers^1^,^4^. Among *Bacillus* strains, *B. licheniformis* WX-02 has been proven as a promising γ-PGA producer^5^. Some researches have been performed to improve γ-PGA production by this strain. For example, nitrate addition improved γ-PGA by 2.3-fold^6^, and the physicochemical stresses such as heat, osmotic and alkaline improved the γ-PGA yield^7^,^8^. Also, over-expression of PgdS (γ-PGA hydrolase) and Glr (glutamic acid racemase) could enhance γ-PGA production^9^,^10^. Meanwhile, the research conducted in our group has revealed that glutamate dehydrogenase RocG played a critical role for the conversion of α-ketoglutarate to glutamic acid, and the function of RocG from *B. licheniformis* WX-02 was different from that of *B. subtilis*^11^. Also, RocG of *B. licheniformis* was confirmed as a NADPH-dependent enzyme during the γ-PGA production^12^.

In recent years, improvement of γ-PGA production in *Bacillus* species has been focused on engineering of the strains’ metabolic pathway^4^. Glutamic acid is the precursor for the synthesis of γ-PGA, and it is mainly converted by α-ketoglutarate in TCA cycle when no glutamic acid is added into the medium. Also, researchers have identified the *pgsB, pgsC* and *pgsA* as essential genes in the γ-PGA biosynthesis, and gene *degU* and *swrA* were reported as positive regulators for the expression of PgsBCA^13^. Based on this basic information, various strategies have been developed to manipulate the metabolic pathway for improving γ-PGA production. For example, genes involving in the byproduct biosynthesis (*sac* responsible for levan synthesis, *lps* responsible for lipopolysaccharide synthesis) were knocked out in *Bacillus amyloliquefaciens* LL3^14^,^15^. Also, Scoffone *et al*., deleted the genes coding for the major γ-PGA degrading enzymes, *pgdS* and *ggt* to enhance the γ-PGA production, and the γ-PGA purity and yield were all increased^16^.

Regulation of cofactor such as NADH and NADPH is another potential method to enhance product production^17^. In general, NADH and NADPH serve as cofactors in various metabolic pathways in bacterial cells. The cofactor NADH plays an important role in the catabolism, and NADPH acts as an essential component for the anabolism. This cofactor has proved as an important cofactor in the synthesis of biopolymers^18^,^19^. NADPH is produced in pentose phosphate pathway (PPP), tricarboxylic acid cycle (TCA) and transhydrogenases system^20^. Xu *et al*. over-expressed NAD kinase in *Corynebacterium crenatum* to improve polyhydroxybutyrate (PHB) yield by 15.7%^21^. In *E. coli*, over-expression of NADPH transhydrogenase UdhA was used as an effective way to improve PHB production^22^. Lim *et al*. improved the PHB production by 41% in the Zwf over-expressed strain^23^.

Based on the previous research, we explore the strategy of cofactor regulation to improve γ-PGA production. Here, we hypothesize that the γ-PGA synthesis of *B. licheniformis* WX-02 could be improved by over-expressing genes related with NADPH generation such as *gnd, zwf, gdh* and *ppnk* from *B. licheniformis* WX-02, *pntAB* and *udhA* from *E. coli* BL21(DE3). The aim of this work is to improve the γ-PGA production by cofactor NADPH generation. The research provides valuable information on a new approach to improve γ-PGA production.

## Results

### Establishment of the recombinant strains

**Figure 1:**
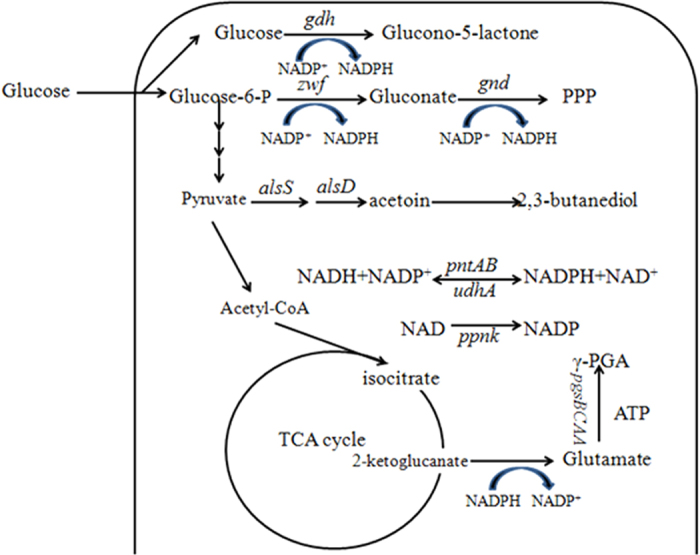
The metabolic pathways of NADPH generation and γ-PGA production in *B. licheniformis* WX-02.

Six enzymes (Zwf, Gnd, Gdh, Ppnk, PntAB and UdhA) related with NADPH production were respectively over-expressed in *B. licheniformis* WX-02 to improve the γ-PGA production (Fig. 1). Here, the construction of expression plasmid pHY-zwf was used as an example (Fig. S1). The plasmid pHY-zwf harboring the P43 promoter (K02174.1), target gene *zwf* (FJ374767.1) and *amyL* terminator (FJ556804.1) was confirmed through PCR verification and DNA sequencing. The plasmid pHY-zwf was then electro-transferred into the native strain *B. licheniformis* WX-02 to construct the recombinant strain *B. licheniformis* WX-zwf. Other recombinants were constructed using the same method and were named WX-gnd, WX-gdh, WX-ppnk, WX-pntAB and WX-udhA, respectively. *B. licheniformis* WX-02 and WX-pHY300 were served as the control strains.

### Production of γ-PGA by the NADPH over-expression strains

**Figure 2:**
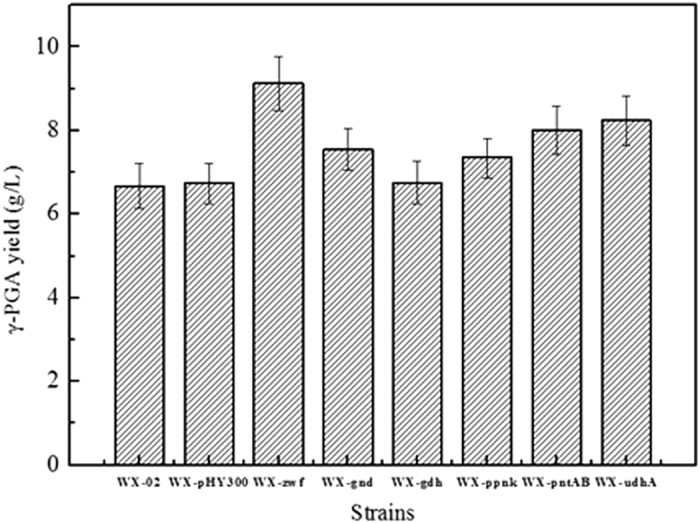
Effects of the over-expression of Zwf, Gdh, Gnd, Ppnk, pntAB and UdhA on the γ-PGA production.

Six recombinant strains (WX-zwf, WX-gnd, WX-gdh, WX-ppnk, WX-pntAB and WX-udhA) and two control strains (WX-02, WX-pHY300) were cultured in γ-PGA production medium to evaluate the effects of NADPH generation on the γ-PGA production. As shown in Fig. 2, WX-zwf showed the highest γ-PGA yield (9.13 g/L) among five recombinant strains, and was 35% higher than that of the control WX-pHY300. The γ-PGA yields of other recombinant strains WX-udhA (8.24 g/L), WX-pntAB (8.01 g/L), WX-gnd (7.55 g/L) and WX-ppnk (7.35 g/L) were 22%, 19%, 12% and 9% higher than that of the control strain WX-pHY300. While the strain WX-gdh did not improve γ-PGA produciton compared to the control strain WX-pHY300. The results indicate that over-expression of the enzymes Zwf, UdhA, PntAB, Gnd and Ppnk in NADPH generation is an effective way to improve γ-PGA production, among which Zwf exhibited the highest improvement of γ-PGA production.

**Figure 3:**
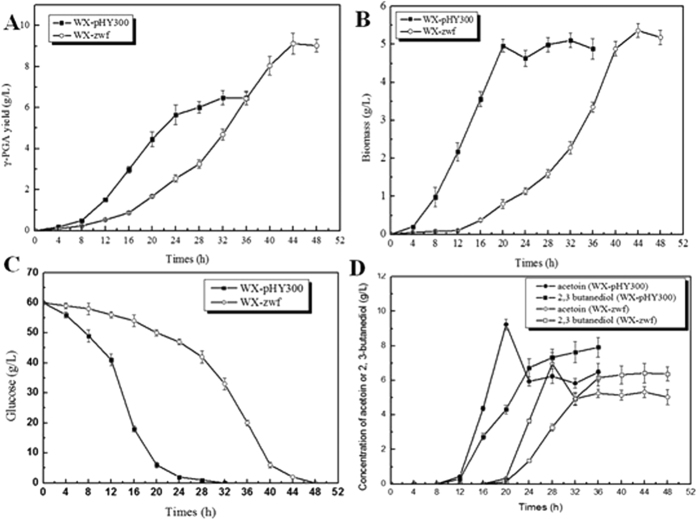
Fermentation process curve of *B. licheniformis* WX-pHY300 and WX-zwf. (**A**) γ-PGA yield; (**B**) Biomass; (**C**) Residue glucose; (**D**) Acetoin and 2,3-butanediol concentrations.

The fermentation of WX-pHY300 and WX-zwf were further performed to determine the time course of γ-PGA production, cell growth, residual glucose, and main byproducts (acetoin and 2,3-butanediol) produced (Fig. 3). As shown in Fig. 3, the control strain WX-pHY300 rapidly entered into the exponential phase after 8-h of lag phase, γ-PGA synthesized rapidly along with rapid cell growth and glucose consumption, these trends were similar to those in previous reports^8^. Compared to the control strain WX-pHY300, the mutant strain WX-zwf had a much longer lag phase (18 h) with a slow growth rate. However, the γ-PGA yield by the WX-zwf strain increased 35%, and the cellular γ-PGA content improved from 1.32 g/g_DCW_ to 1.72 g/g_DCW_ in compared that of the WX-pHY300. The γ-PGA produced by glucose was 0.15 g/g_glc_, 35% increase compared with that of WX-pHY300 (0.11 g/g_glc_). Since WX-zwf had a much longer lag phase (18 h) with a slow growth rate, the production rate of WX-zwf was 0.21 g/L/hr, which has no difference compared with that of WX-pHY300 (0.21 g/L/hr). Also, the glucose uptake rate of WX-zwf was 0.23 g/g_DCW_·h, 36% lower than that of WX-pHY300. Meanwhile, the production of byproducts (acetoin and 2,3-butanediol) decreased 18% in the WX-zwf.

### Transcriptional level of Zwf over-expression strain

**Figure 4:**
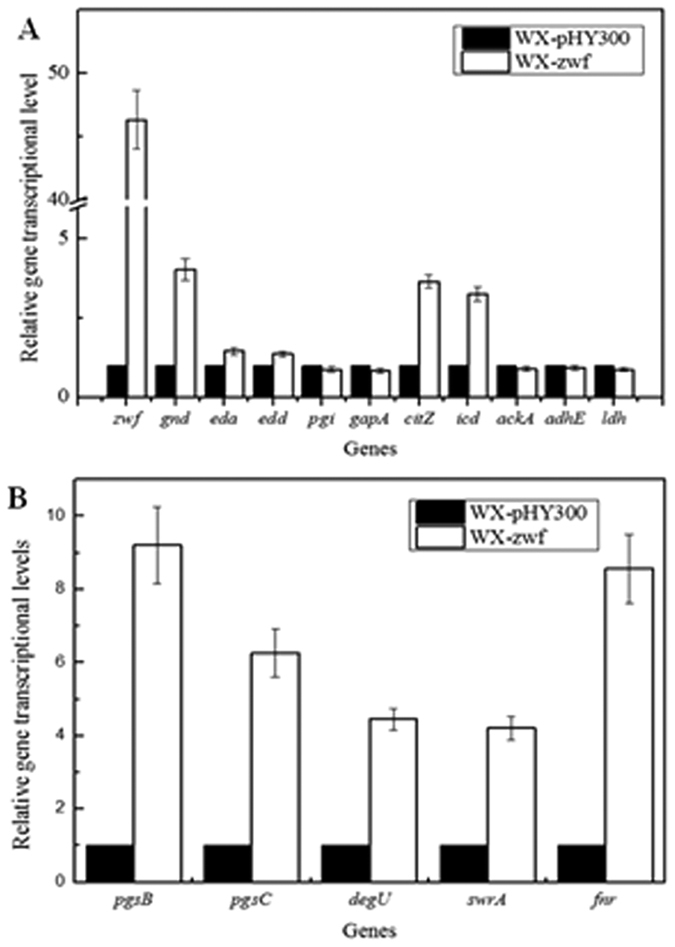
Effects of over-expression of Zwf on the relative transcriptional levels of the genes in the glucose metabolic and γ-PGA biosynthesis.

The transcriptional levels of the genes involved in the γ-PGA biosynthesis and glucose metabolism were determined. As shown in Fig. 4A, the *zwf* transcriptional level in WX-zwf strain was increased by 45.96-fold compared to the control strain WX-pHY300. The *gnd* transcriptional level was also increased (by 3.03-fold) due to the over-expression of Zwf, which might promote NADPH generation as well. Also, 2-dehydro-3-deoxy-phosphogluconate aldolase gene *eda* and phosphogluconate dehydratase gene *edd* in the ED pathway were increased by 0.45-fold and 0.37-fold, and the increase rates were much low than that of *gnd*. Previously, other report using ^13^C labeling indicated that redirecting carbon fluxes to the oxidative pentose phosphate pathway may lead to minor increases of the ED pathway fluxes, which was positively correlated with our results^24^. The transcriptional level of glucose-6-phosphate isomerase gene *pgi* and glyceraldehyde-3-phosphate dehydrogenase gene *gapA* in the glycolysis pathway were decreased because of the strengthening of PPP, while the citrate synthase gene *citB* and isocitrate dehydrogenase gene *icd* transcriptional levels were increased. Meanwhile, acted as the γ-PGA biosynthesis genes, the *pgsB* and *pgsC* transcriptional levels were increased by 8.21-fold and 5.26-fold, respectively. As the essential regulators to activate the γ-PGA biosynthesis, the transcriptional levels of *degU* and *swrA* were up-regulated by 3.45-fold and 3.21-fold (Fig. 4B), which might improve the expression of γ-PGA biosynthetase PgsABC^13^. Also, the improvement of the anaerobic regulator gene *fnr* transcriptional might improve the nitrate reduction and ATP supplement for γ-PGA production^25^. Furthermore, the genes (*ackA, adhE* and *ldh*) responsible for acetate, ethanol and lactate synthesis were all decreased in the Zwf overexpression strain, which indicated that over-expression of Zwf could affect the overflow metabolism.

### Zwf activity of Zwf over-expression strains

**Figure 5:**
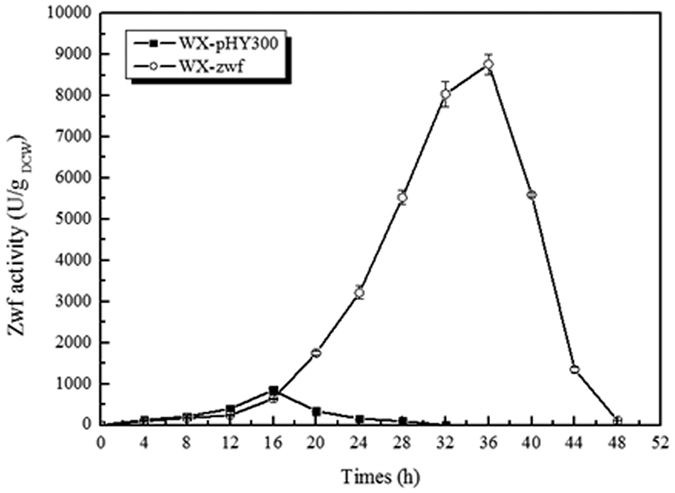
Assay of the activity of Zwf.

The Zwf activity was determined every 4 hours during the γ-PGA production stage. As shown in Fig. 5, Zwf was expressed along with the rapid cell growth. The highest Zwf activity of control strain WX-pHY300 (852 U/g_DCW_) was achieved at the mid-log phase (16 h). The Zwf activity of the recombinant strain WX-zwf was higher than that of WX-pHY300 throughout the fermentation process, with the highest Zwf activity reaching 8762 U/g_DCW_ at 36 h, a 9.28-fold higher than WX-pHY300 strain. The Zwf activity was decreased significantly at the stationary phase (Fig. 5).

### NADPH and NADH production of Zwf over-expression strain

NADPH level was an important factor for γ-PGA biosynthesis, and the highest NADPH content was attained at the exponential phase in each strain, WX-zwf produce 34.49 μmol/g_DCW_ NADPH, 2.36-fold higher than that of WX-pHY300. Meanwhile NADH content in WX-zwf decreased by 27% compared to the control pHY300 strain (Table 1). The ratio of NADPH/NADH was 2.99, a 3.60-fold higher than that of WX-pHY300. Collectively, the results indicated that Zwf over-expression improved the NADPH production, which in turn promoted the glutamic acid and γ-PGA biosynthesis. Meanwhile, NADH plays the vital role in the biosynthesis of 2,3-butanediol^26^,^27^. A decrease of NADH pool in the WX-zwf strains might be reason for the decline of acetoin/2,3-butanediol production.

## Discussion

γ-PGA is an important product with many applications^1^. Metabolic engineering is an effective strategy for the improving the synthesis of target products including γ-PGA. Many tactics have been developed to improve γ-PGA production, including blocking the by-products synthesis pathways and enhancing the glutamic acid synthesis pathways^4^,^14^. However, the strategy of regulating cofactor such as NADPH for improving γ-PGA production has not been attempted, although cofactor regulation in general has been recognized as an effective method for product synthesis. In this report, it was confirmed that NADPH generation was a limiting factor in γ-PGA biosynthesis, over-expression of Zwf was an effective way to improve the γ-PGA production.

Six recombinant strains were constructed to improve the γ-PGA production by NADPH generation, and the γ-PGA yield was in a descending order for those strains as WX-zwf > WX-udhA > WX-pntAB > WX-gnd > WX-ppnk > WX-gdh > WX-pHY300 > WX-02. Over-expression of Zwf showed the most promising outcome. A 12% improvement was obtained in the Gnd over-expressed strain WX-gnd, which was much lower than that of WX-zwf. Since glucose can barely enter cells by Gdh, over-expression of Gdh is not effective to improve NADPH generation and γ-PGA yield. These results were in agreement with the previous research^23^. It should be noted that the Zwf over-expressed strain had a low glucose consumption rate, cell growth rate and γ-PGA biosynthesis rate at the initial phase, there were more rooms to optimize this mutant strains by genetic modification and process engineering.

Among PPP, TCA cycle and the transhydrogenases system pathways, PPP pathway contributes most to NADPH generation. As the first and key gene in PPP, Zwf converts glucose-6-phosphate to 6-phosphogluconate with the conversion of NADP^+^ to NADPH^20^, therefore, over-expression of Zwf increases the NADPH generation, which in turn accelerate the reaction of α-ketoglutarate to glutamate and finally improve the γ-PGA production. Meanwhile, over-expression of Zwf could mainly strengthen the OPP pathway, and strengthening of OPP pathway could promote NADPH generation. Meanwhile, Over-expression of Zwf also weakens the glycolysis pathway, which then reduces the overflow metabolism and byproduct synthesis. Also, the TCA cycle was strengthened in the Zwf over-expression strain, which was beneficial for the γ-PGA production. Collectively, this study shows that the over-expression of Zwf is an effective strategy to improve the yield of target product such as γ-PGA in the NADPH-dependent biotransformation pathway, compared with over-expression of other genes.

Over-expression of Zwf could weaken the glycolysis pathway, which might affect the overflow metabolism. Also, Over-expression of Zwf could convert more NADH and NADP^+^ to NAD^+^ and NADPH respectively, which will decrease the NADH accumulation in the Zwf over-expression strain. Previous reports have proved that NADH plays an important role in the biosynthesis of the acetoin and 2,3-butanediol^26^. The yields of the acetoin and 2,3-butanediol were decreased due to the reduction of the NADH concentration in the strain WX-zwf. Collectively, the increase of the γ-PGA yield was considered due to the improvement of NADPH generation via Zwf over-expression, and the decrease in the synthesis of acetoin/2,3-butanediol byproducts resulted from the reduction of NADH pool.

Served as the two important transcriptional regulation gene for γ-PGA biosynthesis, the increase of expression levels of *degU* and *swrA* could activate the expression of γ-PGA synthetase^13^. Also, Fnr was served as the main anaerobic regulator in *Bacillus*, and the increase of *fnr* transcriptional level might improve the nitrate respiration and ATP synthesis^25^. Our results also indicate that NADPH generation might improve the quorum sensing system and anaerobic metabolism in *B. licheniformis*, which improve the transcriptional levels of *pgsB* and γ-PGA production.

## Conclusion

In this study, six enzymes were respectively over-expressed to improve the γ-PGA production in *B. licheniformis* WX-02. Among the mutant strains, rhe Zwf over-expressed strain WX-zwf showed the highest yield of γ-PGA (9.13 g/L), 35% higher than that of the control strina WX-pHY300. Also, the transcriptional levels of genes responsible for γ-PGA synthesis and Zwf activity were correlated positively with γ-PGA production. The over-expression of Zwf also increased the NADPH concentration as well as the ratio of NADPH/NADH, which led to the improvement of γ-PGA production. The concentration of the main byproduct acetoin/2,3-butanediol was decreased obviously due to the reduction of NADH concentration. Collectively, the results demonstrated that enhanced NADPH generation via over-expression of Zwf can be as an effective strategy to improve the γ-PGA production.

## Materials and Methods

### Bacterial strains and plasmids

The bacterial strains and plasmids used in this study are listed in Table 2. *B. licheniformis* WX-02 (CCTCC M208065) was served as the native strain for the construction of recombinant strains^5^. *E. coli* DH5α was the host strain for plasmid construction. The plasmid pHY300PLK was used as the expression vector^28^.

### Media and culture conditions

LB medium containing (per liter) 10 g peptone, 5 g yeast extract, and 10 g NaCl (pH 7.2) was used as the basic medium. When necessary, ampicillin (50 mg/L) was added to the *E. coli* culture, while tetracycline (20 mg/L) was added to the *B. licheniformis* culture. The seed culture of *B. licheniformis* was prepared in 250 mL flasks containing 50 mL LB medium and incubated at 37 °C in a rotatory shaker (180 rpm) for 10 h until OD_600_ reach 4.0~4.5. The seed was then transferred (3% inoculum ratio) to γ-PGA production medium containing (per liter) 60 g glucose, 10 g sodium nitrate, 10 g sodium citrate, 8 g NH_4_Cl, 1 g CaCl_2_, 1 g K_2_HPO_4_·3H_2_O, 1 g MgSO_4_·7H_2_O, 1 g ZnSO_4_·7H_2_O and 0.15 g MnSO_4_·7H_2_O at pH 7.2^6^. The incubating conditions were the same as those of the seed culture. All the fermentation experiments were performed in three replicates.

### Construction of the over-expression strains

The expression vectors were constructed based on pHY300PLK. The construction of the plasmid containing different genes was exemplified with pHY-zwf (Fig. S1)^28^. Firstly, P43 promoter (K02174.1) from *B. subtilis* 168, the *zwf* gene (52783855) and *amyL* terminator (FJ556804.1) from *B. licheniformis* WX-02 were amplified with the corresponding primers (Table 3). The amplified fragments were ligated by Splicing Overlapping Extension PCR (SOE-PCR) with the primers of P43-F and TamyL-R, and then cloned into pHY300PLK at the restriction sites *Eco*RI and *Xba*I to form the Zwf over-expressing vector, named pHY-zwf. The pHY-zwf vector was then transferred into *B. licheniformis* WX-02 based on the method reported previously^29^. The recombinant strain WX-zwf was confirmed by PCR and plasmids extraction.

The strains over-expressing Gdh, Gnd, Ppnk, PntAB and UdhA were constructed using the same method as that in Zwf, and were respectively named as *B. licheniformis* WX-gdh, WX-gnd, WX-ppnk, WX-pntAB and WX-udhA. The vector pHY300PLK were also transformed into native WX-02 strain to form the control strain WX-pHY300.

### Determination of γ-PGA production, biomass and residue glucose

γ-PGA was determined by HPLC based on the method reported previously^8^. A 7.8 mm × 300 mm TSK Gel G6000 PWXL gel permeation chromatogram column (Tosoh, Tokyo, Japan) was used. The samples were eluted with a mixture of 25 mM sodium sulfate solution: acetonitrile (8:1) with the flow rate of 0.5 mL/min and detected at 220 nm. The γ-PGA yield was quantified based on the standard curve. To determine the cell dry weight, 6 mL distilled water was mixed with 2 mL broth, and the pH of the mixture was adjusted to 2.5 by 6 M HCl. The resulting solution was centrifuged at 12,000 *g* for 10 min, the cell pellets were harvested and dried at 80 °C. The residual glucose in the medium was detected by the SBA-40C bioanalyzer (Academy of science, Shandong, China).

### Determination of acetoin and 2,3-butanediol concentrations

Acetoin and 2,3-butanediol were determined according to the methods reported previously^30^,^31^. In brief, the fermentation broth was mixed with equal volume of ethanol (containing 10 g/L butyl alcohol), centrifuged at 10,000 *g* for 15 min and filtrated through 0.22 μm filter. An Agilent 7890A gas chromatography with the flame ionization detector was then used to identify and quantify acetoin and 2,3-butanediol based on internal standard.

### Analysis of transcription level

Total RNA was extracted by TRIzol^®^ Reagent (Invitrogen, USA) according to previous method^32^. Trace DNA was digested by the Rnase-free DNase I enzyme (TaKaRa, Japan), and RevertAid First Strand cDNA Synthesis Kit (Thermo, USA) was applied to amplify the first stand of cDNA. The primers in Table S1 (seeing Supplementary Material) were used for amplifying the corresponding genes, and 16S rRNA from *B. licheniformis* WX-02 strain was used as the reference gene to normalize the data. The transcriptional levels for genes in the recombinant strain were compared with those of control strain after normalization to the reference gene 16S rRNA. All the experiments were performed in triplicate.

### Assay of the Zwf

The Zwf activity was determined based on the previous method with minor modifications^33^. In brief, the cells were washed three times with cold disruption buffer (100 mM Tris-HCl, 20 mM KCl, 10 mM MgCl_2_, pH 7.5), followed with sonication (pulse: 1 s on; 2 s off; total 4 min) to disrupt cells. The solution with disrupted cells was centrifuged at 12,000 *g* for 20 min. The Zwf activity was determined containing 100 mM Tris-HCl (pH 7.5), 20 mM KCl, 1 mM NADP^+^, 10 mM MgCl_2_ and 5 mM Glucose-6-phosphate. The volume of 2.85 mL mixture was incubated at 30 °C for 5 min, and 150 μL samples were added to start the reactions, and the slope of the A_340_ at the initial reaction (for 0 to 4 min) was calculated, The Zwf activity was calculated as follows, where ΔA was the change of A_340_ per min, V is the reaction volume, ε is the molar extinction coefficient of the NADPH (6.22 × 10^3^ L/(mol·cm)), and b is the light pathlength of the cuvette.


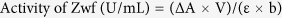


### Determination of NADH and NADPH contents

NADH and NADPH contents were determined by previous method with minor modifications^34^,^35^. For NADH, 20 μL disrupted cell solution were added into plate wells containing 0.1 mL of 0.1 M HEPES (pH 7.5) and 2 mM EDTA, 20 μL of 1.2 mM DCPIP, 10 μL of 20 mM PMS, 25 μL water and 10 μL alcohol dehydrogenase (containing in 0.1 M HEPES (pH 7.5) and 2 mM EDTA). Ethanol (15 μL) was then added to the wells to start the reaction. The decrease of A_600_ was monitored every 30 s for 5 min. The contents were calculated by reference to the standards run concurrently (0~40 pmol NADH in the well). To determine NADPH, G6PDH was dissolved in 0.1 M HEPES (pH 7.5) and 2 mM EDTA, and 30 μL neutralized supernatant were introduced into the plates wells containing 0.1 mL of 0.1 M HEPES (pH 7.5), 2 mM EDTA, 20 μL of 1.2 mM DCPIP, 10 μL of 20 mM PMS, 30 μL water. The reaction was started by the addition of 10 μL G6PDH; the decrease in A_600_ was monitored every 30 s for 5 min. The rates were calculated with the standards, similar to the NADH analysis.

## Additional Information

**How to cite this article:** Cai, D. *et al*. A novel approach to improve poly-γ-glutamic acid production by NADPH Regeneration in *Bacillus licheniformis* WX-02. *Sci. Rep.* **7**, 43404; doi: 10.1038/srep43404 (2017).

**Publisher's note:** Springer Nature remains neutral with regard to jurisdictional claims in published maps and institutional affiliations.

## Supplementary Material

Supplementary Material

## Figures and Tables

**Table 1:** NADPH and NADH concentrations in WX-pHY300 and WX-zwf.

Strains	NADPH concentration (μmol/g_DCW_)	NADH concentration (μmol/g_DCW_)	NADPH/NADH
WX-pHY300	10.25(±0.51)	15.79(±0.97)	0.65(±0.06)
WX-zwf	34.49(±1.62)	11.52(±0.65)	2.99(±0.30)

**Table 2:** The strains and plasmids used in this study.

Strains or plasmids	Description	Source of reference
Strains		
*B. licheniformis* WX-02	Polyglutamate productive strain (CCTCC M208065)	CCTCC
*B. licheniformis* WX-pHY300	*B. licheniformis* WX-02 harboring pHY300PLK	This study
*B. licheniformis* WX-zwf	*B. licheniformis* WX-02 harboring pHY-zwf	This study
*B. licheniformis* WX-gnd	*B. licheniformis* WX-02 harboring pHY-gnd	This study
*B. licheniformis* WX-gdh	*B. licheniformis* WX-02 harboring pHY-gdh	This study
*B. licheniformis* WX-ppnk	*B. licheniformis* WX-02 harboring pHY-ppnk	This study
*B. licheniformis* WX-pntAB	*B. licheniformis* WX-02 harboring pHY-pntAB	This study
*B. licheniformis* WX-udhA	*B. licheniformis* WX-02 harboring pHY-udhA	This study
Plasmids		
pHY300PLK	*E. coli*-*B. licheniformis* shuttle vector, Ap^r^(*E. coli*), Tc^r^ (*E. coli* and *B. licheniformis*)	36
pHY-zwf	pHY300PLK containing P43 promoter, the gene *zwf* and *amyL* terminator	This study
pHY-gnd	pHY300PLK containing P43 promoter, the gene *gnd* and *amyL* terminator	This study
pHY-gdh	pHY300PLK containing P43 promoter, the gene *gdh* and *amyL* terminator	This study
pHY-ppnk	pHY300PLK containing P43 promoter, the gene*ppnk* and *amyL* terminator	This study
pHY-pntAB	pHY300PLK containing P43 promoter, the gene *pntAB* and *amyL* terminator	This study
pHY-UdhA	pHY300PLK containing P43 promoter, the gene *udhA* and *amyL* terminator	This study

**Table 3:** The primers used in this research.

Primer names	Sequence 5′ → 3′^a^	Function
P43-F	CG**GAATTC**TGATAGGTGGTATGTTTTCG	Amplification of P43 promoter
P43-R	TTCATGTGTACATTCCTCTC	
zwf-F	GAGAGGAATGTACACATGAATTGAAAAAAGATCAAATGGAACC	Amplification of *zwf*
zwf-R	AAATCCGTCCTCTCTGCTCTTTTAAAGCGGCCACCAATGAAAG	
gdh-F	GAGAGGAATGTACACATGAAATGTATCCAAGTTTAGAGGG	Amplification of *gdh*
gdh-R	AAATCCGTCCTCTCTGCTCTTTCACCCTTTTCCCGCTTGG	
gnd-F	GAGAGGAATGTACACATGAAATGTGCAATACAATCGGTGTCATAG	Amplification of *gnd*
gnd-R	AAATCCGTCCTCTCTGCTCTTTTAATACCAGTCCGTATGAAACAC	
ppnk-F	GAGAGGAATGTACACATGAAATGATGAAGTTTGCGGTATCG	Amplification of *ppnk*
ppnk-R	AAATCCGTCCTCTCTGCTCTTCTATTCTCCTTTTCCGAT	
pntAB-F	GAGAGGAATGTACACATGAAATGCCACATTCCTACGATTACG	Amplification of *pntAB*
pntAB-R	AAATCCGTCCTCTCTGCTCTTTTAAAACAGGCGGTTTAAACC	
udhA-F	GAGAGGAATGTACACATGAAATGCGAATTGGCATACCAAGAG	Amplification of *udhA*
udhA-R	AAATCCGTCCTCTCTGCTCTTTTACAGAGCTTTCAGGATTGCATC	
TamyL-F	AAGAGCAGAGAGGACGGATTT	Amplification of *amyL* terminator
TamyL-R	GC**TCTAGA**GCCGCAATAATGCCGTCGCACTG	
pHY-F	GTTTATTATCCATACCCTTAC	Verification primers for the expression vectors
pHY-R	CAGATTTCGTGATGCTTGTC	

^a^Restriction sites highlight in bold. Underline stands for the overlap region for splicing by overlapping extension PCR (SOE-PCR).
