# Supplementary material for: A novel approach to improve poly-γ-glutamic acid production by NADPH Regeneration in Bacillus licheniformis WX-02
Source: Sci Rep. 2017 Feb 23;7:43404. doi: 10.1038/srep43404 (PMC5322528; doi:10.1038/srep43404)
Supplement: Supplementary Material [file srep43404-s1.pdf]

A novel approach to improve poly-γ-glutamic acid production by NADPH Regeneration in *Bacillus licheniformis* WX-02

Dongbo Cai [a], Penghui He [a], Xingcheng Lu [a], Chengjun Zhu [a], Jiang Zhu [a], Yangyang Zhan [a], Qin Wang [a], Zhiyou Wen [b,c], Shouwen Chen [a*]

[a] *Hubei Collaborative Innovation Center for Green Transformation of Bio-Resources*, *College of Life Sciences, Hubei University, Wuhan 430062, China*

[b] *College of Food Science and Technology, Huazhong Agricultural University, Wuhan 430070, China*

[c] *Department of Food Science and Human Nutrition, Iowa State University, Ames, Iowa 50011, United States*

*Corresponding author. Shouwen Chen

Tel./fax.: +86 027-88666081.

E-mail address: mel212@126.com (S. Chen).

Postal address: 368 Youyi Avenue, Wuchang District, Wuhan 430062, Hubei, PR China

Table S1 The primers used for RT-qPCR in this research

Genes	Forward	Reverse
16S rRNA	TCAGCTCGTGTCGTGAGAT	CGATCCGAACTGAGAACAG
zwf	GCCGATCAAGCTCGACTACT	CCATAGAGCCCGCCTCATAGT
gnd	AGCTTGAAGATTTCGTACAG	TCGCCGCCGGAAATGCCGAT
pgsB	ATCAAAAGAAAGCCGCAAGG	AGTCGGTCCCATCACATCC
pgsC	GCTGCTCGTTCTTTTTGTC	CGTACTTCCAAGCGTAATCG
degU	GATCAGCGTATCGGCATCC	TTTGAGGTAGTGGCCGAAGG
swrA	GCTCATCCAAAGCGTCACAT	CGTTTTCCGCTACAAAGGCAG
fnr	TGACGTCCGATGGCAAAGAA	AAACTCAAACGTCAGCGCAC

Fig S1: The construction procedure of the over-expression vector pHY-zwf.

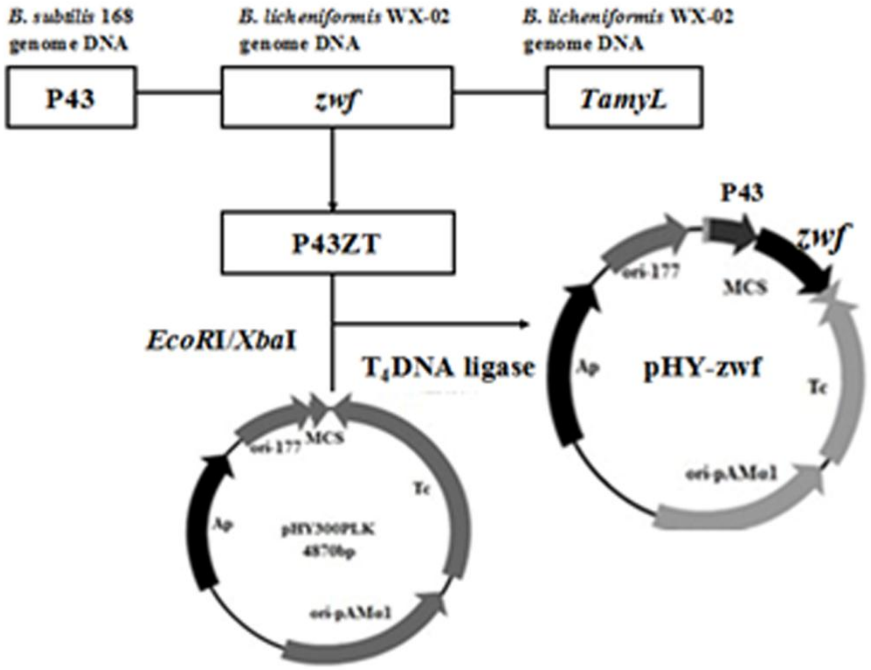

Fig. S1
